# Pheochromocytoma triggered by coronavirus disease 2019: a case report

**DOI:** 10.1186/s13256-022-03378-8

**Published:** 2022-06-10

**Authors:** Hoda Naghshineh, Amirhossein Hasanpour, Naghmeh Ziaei, Mahmoud Sadeghi, Neda Meftah

**Affiliations:** 1 Clinical Research Development Unit of Rouhani Hospital, Babol University of Medical Sciences, Ganjafroz Street, Babol, I. R. of Iran; 2 Student Research Committee, Babol University of Medical Sciences, Babol, Iran

**Keywords:** Pheochromocytoma, Coronavirus disease, MEN-2 syndrome, Cardiomyopathy, Pheochromocytoma, COVID-19, Case report

## Abstract

**Background:**

Coronavirus disease 2019 is an infectious disease with many presentations, and many of its effects on the human body are still unknown. Pheochromocytoma is a neuroendocrine tumor that may occur sporadically or be a manifestation of a hereditary disease line multiple endocrine neoplasia type 2.

**Case presentation:**

In this study, we report a case of an Iranian patient infected with coronavirus disease 2019, causing unusual presentations of pheochromocytoma, including myocarditis and cerebrovascular involvement.

**Conclusions:**

We discovered a case of pheochromocytoma as an unusual presentation of COVID-19. In further investigations we also discovered thyroid medullary carcinoma and at the end MEN 2 syndrome was diagnosed. After proper treatment many symptoms were eliminated.

## Background

At the end of 2019, an acute respiratory pneumonia known as coronavirus disease 2019 (COVID-19) caused by severe acute respiratory syndrome coronavirus 2 (SARS-CoV-2) had emerged in China. Despite having high transmissibility and affecting millions of people worldwide, many aspects of its effects on human body are still unknown [1].

Pheochromocytoma as a neuroendocrine tumor is known to cause overproduction of catecholamines, which can various consequences in the body [2]. Cardiovascular symptoms are one of the most prevalent and complicated effects of pheochromocytoma. Studies have suggested that cardiomyopathies can be a result of catecholamine intoxication. Moreover, several studies have reported that the cardiomyopathies caused by pheochromocytoma can be reversible if diagnosed and treated properly [2–6].

Although there are many articles on COVID-19 or pheochromocytoma separately, there are not enough data to evaluate the relationship between these two major diseases. However, some studies indicate that there is evidence of direct and indirect effects of SARS-COV-2 on adrenal cells such as hypocortisolism, adrenal insufficiency, and hypothalamic-pituitary-adrenal axis dysfunction [7, 8].

In this case report, we present the case of a patient with heart failure and cardiomyopathy admitted to hospital with dyspnea and headache, who then developed ischemic infarctions. He was diagnosed with pheochromocytoma following COVID-19 infection.

## Case presentation

A 32-year-old Iranian man with headache and dyspnea was admitted to Rouhani Hospital. On initial assessment, his systolic blood pressure was 170/100 mmHg. About a week prior to his admission, he had an open appendectomy and his postsurgery systolic blood pressures were in the range of 140–180 mmHg. In this hospitalization, COVID-19 was suspected owing to his flu-like symptoms (myalgia, headache, and dyspnea). Therefore, computed tomography (CT) and polymerase chain reaction (PCR) were performed. The results revealed suspicious CT scan and positive PCR.

Because of dyspnea and hypertension, an electrocardiogram (ECG) was performed, which found diffuse ST-segment elevation; hence, he underwent echocardiography. On further examinations, he had positive serum troponin, about 35% ejection fraction (EF), and ventricular wall and papillary muscles thickness. Transesophageal echocardiography (TEE) also showed moderate-to-severe systolic dysfunction and significant diastolic dysfunction in the left ventricle, but no clot was found. During hospitalization, we observed persistent high blood pressure that could not be controlled using three drugs. Additionally, he had sweating, high pulse rate, and abnormal liver function tests. At this time, he was receiving anticoagulant (heparin), antiviral therapy (remdesivir), and antibiotics (targocid and meropenem) in addition to the routine treatments.

Four days after admission, the patient’s condition worsened. Neurological examination revealed right-sided hemiplegia and dysarthria. However, funduscopic examination was normal. In addition, his level of consciousness had gradually decreased insofar as he could not obey commands and make meaningful eye contact, but he could move his unaffected limbs. Imaging [brain CT and magnetic resonance imaging (MRI) scans] findings were compatible with the diagnosis of ischemic infarction. Though some brain CT findings were suggestive of cortical vein thrombosis, brain MRI with contrast, brain images, and MR venography could not confirm this diagnosis. The MRI demonstrated multiple bilateral acute ischemic changes with lesions in the right high-parietal cortex as well as left temporal and basal ganglia regions being more prominent. Brain and cervical MR angiography were unremarkable.

Considering the diagnosis of cardioembolic infarct, intravenous anticoagulant therapy with heparin was started. This treatment was discontinued after 2 days because some hemorrhagic changes were seen on imaging studies. His systolic blood pressure was 240 mmHg at this stage.

Two days later, he regained consciousness, and his weakened limbs started recovering gradually.

After 6 days of having high blood pressure, episodic headaches, and sustained tachycardia, pheochromocytoma was strongly suspected.

**Fig. 1:**
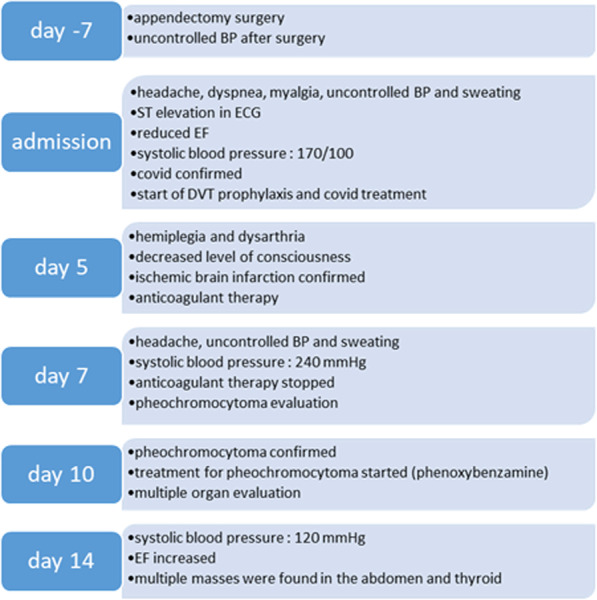
Overview of the patient hospitalization process

In Fig. 1, we provide an overview of the hospitalization course of the patient.

Urinary metanephrine (MN), normetanephrine (NMN), and vanillylmandelic acid (VMA) were collected in a 24-h period and were positive, leading to diagnosis of pheochromocytoma.

In addition, multiple masses were found in the abdomen and thyroid. Phenoxybenzamine was prescribed, his systolic blood pressure came down to 120 mmHg, and 2 weeks later, his EF was 45–50%. The patient was considered a candidate for surgery to treat pheochromocytoma, but owing to rapid EF, symptom improvement, and the recent CVA, it was postponed 2 months.

Considering a familial syndrome, a survey was done to detect possible familial cases. In this case report, the patient’s mother had high blood pressure and positive pheochromocytoma.

Regarding all signs and symptoms (medullary thyroid carcinoma and pheochromocytoma), the final diagnosis was multiple endocrine neoplasia type 2 (MEN 2) syndrome.

### Laboratory and clinical findings

At first, the patient was admitted with dyspnea and headache; hence, inflammatory tests, CT scan, and PCR were done to assess the patient for COVID-19. As mentioned previously, CT scan was suspicious and PCR was positive for COVID. However, as were unable to justify all of the symptoms with COVID-19, we requested hematologic, cardiac, and hepatic tests. These laboratory tests revealed normal inflammation markers [erythrocyte sedimentation rate (ESR) and C-reactive protein (CRP)] and hematologic tests (CBC diff), and hepatic markers [alanine aminotransferase (ALT), alkaline phosphatase (ALP), aspartate aminotransferase (AST), prothrombin time (PT), partial thromboplastin time (PTT), and total and direct bilirubin] were slightly increased, but cardiac markers [troponin and pro-B-type natriuretic peptide (BNP)] were high and procalcitonin was positive. At this stage, myocardial infarction or cardiomyopathy was suspected; thus, the transthoracic and TEE were performed. EF was about 35%, and the ventricular walls were thicker than normal. Furthermore, severe systolic and moderate diastolic dysfunctions were detected on TEE, but no clot was found.

Laboratory tests were rechecked and confirmed the next day. After that, D-dimer was requested, and it was highly positive. Rheumatologic markers were checked during hospitalization, but none was positive.

Then, 6 days later, after clinical suspicion of pheochromocytoma, urinary catecholamines (MN, NMN, and VMA) were tested, and all of them returned positive for pheochromocytoma.

On spinal and abdominal CT scans with contrast, multiple masses were observed in the abdomen, and multiple nodules were seen on thyroid ultrasonography. Additionally, lymphadenopathy was significant in the left thyroid gland.

Biopsy was done from thyroid nodules, and medullary carcinoma was detected.

All laboratory test results can be found in Table 1.

**Table 1:** Patient’s laboratory test results. We briefed patient’s lab tests in three time points. Admission time, average amounts during treatment and discharge time to understand the treatment process

No.	Test	Admission time	During Treatment	Discharge time	Normal range (unit)
1.	WBC	13,400	17,575	15,400	4.5–11.0 × 10^9^ (cells/L)
2.	RBC	5/33	5/15	4/56	4.7–6.1 × 10^6^ (cells/μl)
3.	HB	15/7	15/09	13/7	13.5–17.5 (g/dl)
4.	HTC	45	43/20	37/9	41–50 (%)
5.	PLT	208,000	345,750	397,000	150,000–450,000 (/μl)
6.	PT	12	12/22	12	11–13.5 (seconds)
7.	PTT	26	53/64	31	25–35 (seconds)
8.	BUN	36	31/08		7–20 (mg/dl)
9.	Cr	1	0/98		0.6–1.2 (mg/dl)
10.	Na	144	132/64		135–145 (mEq/l)
11.	K	3/6	4/22		3.6–5.2 (mmol/l)
12.	Total bilirubin	1/4	1/18		Up to 1.2 (mg/dl)
13.	Direct bilirubin	0/4	0/36		Up to 0.3 (mg/dl)
14.	AST	94	75/60		5–40 (IU/L)
15.	ALT	88	99/00		7–56 (IU/L)
16.	ALP	203	294/60		44–147 (IU/L)
17.	Amylase	56	88/40		30–110 (IU/L)
18.	Lipase	61	65/80		10–140 (IU/L)
19.	U/C		Neg		Neg
20.	ESR	5	6/75		0–15 (mm/hour)
21.	CRP	7	10/00		Up to 10 (mg/L)
22.	BS		191/50		72–99 (mg/dl)
23.	PCT	2/8	6/00		Up to 0/5 (ng/ml)
24.	Lupus anticoagulant		33		30–45 (seconds)
25.	D-dimer		856		Up to 500 (ng/ml)
26.	Troponin	1/8			Up to 0/32 (ng/ml)
27.	Pro-BNP	11114			Up to 125 (ng/ml)
28.	IL-6	9/7			0–16.4 (pg/ml)
29.	VMA		111		2–7 (mg/24 hours)
30.	MET 24H		11,463		12–60 (pg/ml)
31.	NMET 24H		14,038		18–111 (pg/ml)
32.	TSH			3/7	0.5–5.0 (mIU/L)
33.	VIT D			17	20–50 (ng/ml)
34.	Ca			11/2	8.6–10.3 (mg/dl)
35.	P			2/2	3.4–4.5 (mg/dl)
36.	Urine free cortisol		715		10–100 (mcg/24 h)
37.	HBS Ag		0/2		Up to 0/89 (ratio)
38.	C3		152		90–180 (mg%)
39.	C4		31		10–40 (mg%)
40.	CH50		99		50–150 (%)
41.	ANA		Neg		Up to 1/80 (titer)
42.	HCV Ab		Neg		Not detected
43.	cANCA		12		Up to 20 (IU/ml)
44.	pANCA		4		Up to 20 (U/ml)
45.	Anti-beta-2 glycoprotein (IgG)		0/2		Up to 10 (U/ml)
46.	Anti-dsDNA		3		Up to 20 (IU/ml)
47.	CPK		79		20–200 (U/L)
48.	Aldolase		3/1		Up to 7.6 (U/L)
49.	Anticardiolipin (IgG)		0/5		Up to 10 (U/μl)
50.	AMA-M2		Neg		Not detected
51.	Mi-2		Neg		Not detected
52.	Ku		Neg		Not detected
53.	Plasma aldosterone		128		
54.	Plasma renin activity		2/4		0.6–4.3 (ng/ml/hour)
55.	PAC/PRA		5/3		

*WBC* White Blood Cell, *RBC* Red Blood Cell, *HB* Hemoglobin, *HTC* Hematocrit, *PLT* Platelets, *PT* Prothrombin Time, *PTT* Partial Thromboplastin Time, *BUN* Blood Urea Nitrogen, *Cr* Creatinine, *Ma* sodium, *K* Potassium, *Bill T* Total Bilirubin, *Bill D* Direct Bilirubin, *AST* Aspartate transaminase, *ALT* Alanine aminotransferase, *ALP* Alkaline Phosphatase, *U/C* Urine Culture, *ESR* Erythrocyte Sedimentation Rate, *CRP* C-reactive protein, *BS* Blood Sugar, *PCT* Procalcitonin Test, *Pro-BNP* Pro-Brain Natriuretic Peptide, *IL-6* Interleukin-6, *VMA* Vanillylmandelic Acid, *MET 24H* Metanephrine urine 24h, *NMET 24H* normetanephrine urine 24h, *TSH* thyroid stimulating hormone, *VIT D* Vitamin D 25 OH, *Ca* Calcium, *P*: phosphorous, *HBS Ag* Hepatitis B surface antigen, *C3 and C4* Complement C3 and C4, *CH50* total hemolytic complement, *ANA* antinuclear antibodies, *HCV Ab* hepatitis C virus Antibody, *C.ANCA* central antineutrophil cytoplasmic antibodies, *P.ANCA* Perinuclear anti-neutrophil cytoplasmic antibodies, *Anti ds DNA* The anti-double stranded DNA, *CPK* Creatine phosphokinase, *AMA-M2* antimitochondrial antibody- M2, *PAC/PRA* plasma aldosterone concentration to plasma renin activity ratio

## Discussion

COVID-19 is a viral disease caused by severe acute respiratory syndrome coronavirus 2 (SARS-CoV-2) influencing the body in various ways, making it difficult to diagnose and detect. Patients with preexisting diseases are at higher risk of morbidity and mortality [9, 10].

There is little evidence to prove the relationship between COVID-19 infection and pheochromocytoma, but it was assumed that in the present case that the exacerbation of the disease was related to COVID-19 infection.

Pheochromocytoma is a rare disease, manifesting with paroxysmal or sustained hypertension, attacks of palpitations, tremors, perspiration, headache, and anxiety.

Cardiovascular manifestations such as acute heart failure, arrhythmias, angina pectoris, myocardial infarction, and dilated cardiomyopathy are considered rare [6]. It is critical to diagnose pheochromocytoma because the consequences can usually be cured through surgery or medical treatment [11, 12].

In this case report, a 32-year-old man presented first with headache, dyspnea, and low EF. The symptoms were suspicious for a recent infection, and because he had low EF and myocardial thickness on echocardiography, myocarditis was considered. Nevertheless, owing to positive troponin as well as ST- and T-segment elevation, myocardial infarction could not be ruled out. However, during treatment, he developed sustained hypertension and multiple ischemic strokes, raising suspicion for other diseases.

Owing to the multiorgan involvement, rheumatologic diseases were considered, too. In laboratory tests, all rheumatologic markers were negative.

Hypertension, congestive heart failure, and cerebrovascular events are reported consequences of pheochromocytoma. In addition, cerebral ischemia and stroke, though rare, can occur [5].

The most unusual aspect of our patient’s condition related to his pheochromocytoma was cardiomyopathy and brain infarctions in the absence of atrial fibrillation or clot. His first presentations were headache and dyspnea.

A cerebrovascular accident is a rare manifestation of pheochromocytoma, and it can occur in several conditions. Hypertension, embolization associated with dilated cardiomyopathy, and vascular spasms induced by sympathomimetic agents as well as, in many cases, focal neurologic deficits are reversible [3].

In patients presenting with dilated cardiomyopathy and focal cerebral symptoms with no exact origin [no clot on TEE or atrial fibrillation (AF) rhythm on ECG, vascular diseases] while having persistent hypertension, pheochromocytoma should be evaluated [5].

Nevertheless, the lack of an exact origin for cerebral infarcts (no clot on TEE or AF rhythm on ECG) in these patients cannot rule out the cardiac source, especially in those with low fractional shortening of the left ventricle [5].

The noncardiac symptoms of the patient in the current report included myalgia, headache, dyspnea, and acute multiple brain infarctions, of which the latter could be attributed to a probable cardiac source or hypertension crisis [3, 5].

A necessary step in the diagnosis of this disease is to evaluate catecholamine levels in the blood. Classic tests assess plasma catecholamines, including urinary MN, NMN, and VMA [13].

Inflammatory markers and leukocytes increase in the presence of chronic excess of catecholamines [3]. Further, in the present report, we evaluated catecholamines and inflammatory markers in the patient, and found that the catecholamines were high and positive, but inflammatory markers (ESR and CRP) were negative. Leukocyte count was 16,910 cells per liter (higher than normal range).

In case of early diagnosis and proper treatment, pheochromocytoma is one of the reversible causes of cardiomyopathy [2]. However, in our case, the patient’s signs and symptoms were relieved after 1 month of treatment with phenoxybenzamine. His EF was normal (55%) after treatment, and his blood pressure decreased. Further, his neurological symptoms related to multiple infarctions improved.

## Conclusion

Considering that not all effects of COVID-19 on the human body are known yet, the simultaneous presence of COVID-19 and pheochromocytoma in a person who didn’t have any specific symptoms so far, may indicate that the COVID-19 has triggered pheochro-mocytoma. Also, thromboembolism and decreased EF are rare manifestations of pheo-chromocytoma that can be a result of or triggered by inflammatory and thrombogenic conditions caused by COVID-19.

## Data Availability

The datasets supporting the conclusions of this article are included within the article. The datasets used during the current study are available from the corresponding author on reasonable request.
